# The BiP Cochaperone ERdj4 Is Required for B Cell Development and Function

**DOI:** 10.1371/journal.pone.0107473

**Published:** 2014-09-15

**Authors:** Jill M. Fritz, Timothy E. Weaver

**Affiliations:** Perinatal Institute, Section of Neonatology, Perinatal and Pulmonary Biology Cincinnati Children’s Hospital Medical Center and the University of Cincinnati College of Medicine, Cincinnati, Ohio, United States of America; University of Geneva, Switzerland

## Abstract

ERdj4 is a BiP cochaperone regulated by the unfolded protein response to facilitate degradation of unfolded and/or misfolded proteins in the endoplasmic reticulum. As the unfolded protein response plays a critical role in B cell maturation and antibody production, ERdj4 gene trap mice were generated to determine if this chaperone was required for B cell homeostasis. Homozygosity for the trapped allele resulted in hypomorphic expression of ERdj4 in bone marrow cells and abnormal development of hematopoietic lineages in the bone marrow. The number of myeloid cells was increased, while the number of erythroid and B lymphoid cells was reduced in ERdj4 gene trap mice compared to controls. An intrinsic B cell defect was identified that decreased survival of B cell precursors including large and small pre-B, and immature B cells. Consistent with impaired B lymphopoiesis, the number of mature follicular B cells was reduced in both the bone marrow and spleen of ERdj4 gene trap mice. Paradoxically, unchallenged ERdj4 gene trap mice showed non-specific hypergammaglobulinemia and gene trap B cells exhibited increased proliferation, survival and isotype switching in response to LPS stimulation. Although ERdj4 gene trap mice responded normally to T cell-independent antigen, they failed to mount a specific antibody response to T cell-dependent antigen in vivo. Collectively, these findings demonstrate that the chaperone activity of ERdj4 is required for survival of B cell progenitors and normal antibody production.

## Introduction

The development and function of B lymphocytes requires activation of the inositol-requiring enzyme 1 alpha (IRE1α) signaling branch of the unfolded protein response (UPR). IRE1α is localized in the endoplasmic reticulum (ER) membrane and activated when the ER chaperone BiP is recruited away from the luminal domain of IRE1α to unfolded/misfolded substrates. The endoribonuclease activity of IRE1α splices an intron from the mRNA of X-box-protein 1 (XBP1) resulting in translation of a transcription factor that upregulates genes associated with ER biogenesis, protein folding and ER-associated degradation (ERAD) [1], [2].

During early B cell development, IRE1α is required at the pro-B cell stage for immunoglobulin heavy chain gene rearrangement [3]. Consistent with these findings, spliced XBP1 is upregulated in pro-B cells [4]. XBP1 splicing also occurs in transitional and mature B cells in the spleen following stimulation of the B cell receptor (BCR) [5]. During plasma cell differentiation, XBP1 is upregulated to promote ER expansion and increase protein folding, glycosylation and trafficking [6]–[9]. Although B cells deficient in XBP1 generate normal numbers of plasma cells, their ability to secrete antibodies is impaired [10]–[12]. Thus, IRE1α/XBP1 is not required for plasma cell differentiation, but rather, to increase the secretory apparatus necessary for immunoglobulin synthesis.

ER-localized DnaJ 4 (ERdj4) is a downstream effector of the IRE1α/XBP1 pathway [9]. ERdj4 belongs to the HSP40 family of cochaperones, which function to stimulate the ATPase activity of BiP, leading to a conformational change that stabilizes client interaction [13]. Functional domains of ERdj4 include a J domain that associates with BiP and a glycine/phenylalanine-rich region that likely interacts with unfolded or misfolded substrates. ERdj4 facilitates the removal of newly synthesized unfolded/misfolded protein substrates from the ER lumen by associating with the ERAD machinery via a poorly understood mechanism [14], [15]. Although ERdj4 expression is highly upregulated in response to ER stress [13], [16], recent studies revealed an unanticipated role for ERdj4 in growth, development and metabolism. Hypomorphic expression of ERdj4 in mice resulted in perinatal lethality associated with growth restriction and hypoglycemia, while surviving adult mice were glucose intolerant and hypoinsulinemic, with defects in the pancreatic β-cell secretory pathway [17].

In the current study, we investigated the role of ERdj4 in hematopoiesis. ERdj4 gene trap (ERdj4^gt/gt^) mice exhibited abnormal numbers of myeloid, erythroid and B lymphoid cells in the bone marrow. Further analyses of B cell development revealed an intrinsic defect that reduced survival of large and small pre-B, and immature B cells in ERdj4^gt/gt^ mice. Consistent with these findings, mature recirculating B cells were decreased in the bone marrow and spleen of ERdj4^gt/gt^ mice. Unexpectedly, basal immunoglobulins were increased in ERdj4^gt/gt^ mice in association with enhanced class switch recombination in vitro; however, ERdj4^gt/gt^ mice failed to mount a specific antibody response to T cell-dependent antigen. Collectively, these data indicate that the chaperone activity of ERdj4 is required for normal development of hematopoietic lineages and function of B lymphocytes.

## Materials and Methods

### Mice

Hypomorphic ERdj4 gene trap mice were generated from an embryonic stem cell line harboring a gene trap cassette in intron 1 (Bay Genomics), as previously described [17]. All mice used in these experiments were 6–16 weeks old in the C57BL/6 genetic background unless otherwise specified. Mice were housed in a pathogen-free barrier facility and experiments were conducted with approval from the Cincinnati Children’s Hospital Medical Center’s Animal Care and Use Committee (Permit number: 3E02017). To minimize suffering, blood collection and injections were performed using isoflurane anesthesia, and mice were sacrificed by carbon dioxide inhalation.

### Tissue harvest and cell isolation

Mouse femurs and tibias were harvested, trimmed and flushed with complete RPMI media (10% FBS, 100 U/ml penicillin, 100 µg/ml streptomycin and L-glutamine) using a 3 cc syringe and a 21 gauge (g) needle to release bone marrow. Single cell suspensions were obtained by repeatedly passing the cells through a syringe. Mouse spleens were harvested and placed on a 100 µm nylon mesh strainer in a 50 mL conical tube. Spleens were gently massaged with the blunt end of a plunger from a 3 cc syringe and the strainer was rinsed with complete RPMI media to release cells.

### Flow cytometry

Bone marrow and spleen cells (1×10^6^ cells/100 µl) were incubated in staining buffer (1X PBS, 1% FBS, 0.05% NaN_3_) containing CD16/32 Fc-blocking antibody (Biolegend, 93) for 30 minutes at 4°C and then stained with fluorescently-labeled antibodies for 30 minutes at 4°C. Data was acquired on the LSRFortessa or LSR II flow cytometers (BD Biosciences) and analyzed with FlowJo software (TreeStar, Inc.). The mouse-specific monoclonal antibodies used for flow cytometry are listed in Table S1.

### Bone marrow transplantation

Mice were exposed to two doses of irradiation and intravenously transplanted with bone marrow (5×10^6^ cells/200 µl) by the Cincinnati Children’s Comprehensive Mouse and Cancer Core. Bone marrow B cells were analyzed 16 weeks after transplantation by flow cytometry.

### Quantitative RT-PCR

Total RNA was extracted from cells using the RNeasy Plus Mini Kit (Qiagen) and cDNA was synthesized using iScript cDNA Synthesis Kit (Bio-rad). Quantitative RT-PCR was performed with 25 ng of cDNA per reaction on the StepOnePlus system with TaqMan assays (Applied Biosystems) for mouse ERdj4 (Mm01622956_s1), eukaryotic 18S rRNA (endogenous control) and β-actin (endogenous control, Mm00607939_s1). Relative quantitation was determined using the SDS software (Applied Biosystems).

### Class switch recombination *in vitro*

Resting B cells were isolated from splenocytes using the B cell isolation kit (Miltenyi Biotec) per manufacturer’s instructions. Isolated B cells were pre-incubated in 1 µM carboxyfluorescein succinimidyl ester (CFSE) for 10 min at 37°C and cultured at 1×10^6^ cells/mL in complete RPMI media containing 50 µM β-mercaptoethanol in the presence of LPS (*E. coli* 055:B5, Sigma-Aldrich). After 72 hours, B cells were permeabilized and washed with BD Cytofix/Cytoperm Kit (BD Biosciences) per manufacturer’s instructions before staining for IgG3 using the methods described above. Viability of B cells was assessed by flow cytometry using the Fixable Viability Dye eFluor 780 (Ebioscience).

### B cell proliferation *in vivo*

To assess bone marrow B cell proliferation, mice were intraperitoneally injected with BrdU (2 mg) 12 hours before sacrifice. For splenic B cell proliferation, mice were intraperitoneally injected with BrdU (1 mg) 12 and 24 hours before sacrifice. Incorporation of BrdU in bone marrow and splenic B cells was quantitated by flow cytometry using the BrdU Flow Kit (BD Pharmigen).

### B Cell function *in vivo*

Basal immunoglobulins were measured in the serum of adult mice using the mouse IgM, IgG or IgA Ready-SET-Go ELISAs (Ebioscience) or the mouse IgE ELISA MAX™ Deluxe (Biolegend). To assess antigen-specific immunoglobulin production, mice were intraperitoneally injected with either ^36^TNP-Ficoll (Biosearch Technologies, 50 µg) or ^21^TNP-CGG (Biosearch Technologies, 100 µg) in Imject Alum adjuvant (Pierce, 1∶1 antigen to adjuvant ratio). Mice were retroorbitally bled before challenge (d0) and on days 7, 14 and 21 after challenge. Microplates were coated with ^18^TNP-BSA (10 µg/mL, Biosearch Technologies) in carbonate coating buffer (Biolegend) overnight at 4°C. After 4 washes in 1X PBS with 0.05% Tween (PBST), plates were blocked in 10% FBS for 1 hour at room temperature and washed in PBST. Serum was diluted in blocking buffer (^21^TNP-CGG, 1∶250; ^36^TNP-Ficoll, 1∶200) and incubated for 1 hour at room temperature. Plates were washed in PBST and incubated with HRP-conjugated mouse IgG or IgM antibody (Bethyl Laboratories). After PBST washing, samples were developed with the 3, 3′, 5, 5′ tetramethyl benzidine (Biolegend) and analyzed at an optical density of 450 nm using a microplate reader.

### Statistical analysis

All experiments were performed at least twice and analyzed by a two-tailed Student’s *t* test using GraphPad Prism software. Data were presented as mean ± SEM with *p≤0.05, **p≤0.01 or ***p≤0.001 significance.

## Results

### ERdj4 deficiency alters hematopoiesis

**Figure 1:**
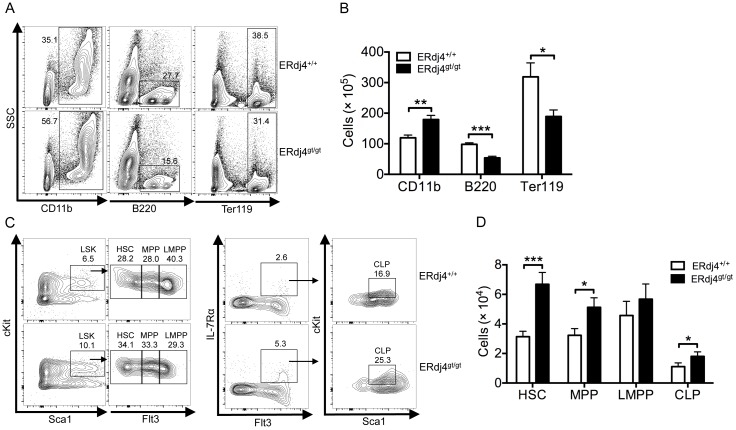
ERdj4 deficiency alters hematopoiesis. (A) Representative contour plots from flow cytometric analyses of myeloid (CD11b^+^), B lymphoid (B220^+^) and erythroid (Ter119^+^) cells in mouse bone marrow. Numbers indicate frequency of the gated population for each genotype. (B) Absolute number of myeloid, B lymphoid and erythroid cells in mouse bone marrow. *n* = 6–8 mice/genotype. (C) Left panel, representative contour plots from flow cytometric analyses of hematopoietic progenitor cells (LSK, Lin^−^cKit^+^Sca1^+^) in mouse bone marrow. LSK cells were lineage-negative (NK1.1^−^Gr.1^−^Ter119^−^CD11b^−^B220^−^CD3^−^) and further defined by Flt3 expression into hematopoetic stem cells (HSCs, Flt3^−^), multipotent progenitors (MPPs, Flt3^low^) and lymphoid-primed MPPs (LMPPs, Flt3^high^). Right panel, representative contour plots from flow cytometric analyses of common lymphoid progenitors (CLPs, Lin^−^IL-7Rα^+^Flt3^+^cKit^low^Sca1^low^) in mouse bone marrow. Numbers indicate frequency of the gated population for each genotype. (D) Absolute number of HSCs, MPPs, LMPPs and CLPs in mouse bone marrow. *n* = 9–11 mice/genotype.

ERdj4 mRNA was significantly reduced in the bone marrow of ERdj4^gt/gt^ mice (Figure S1A), consistent with previous findings that insertion of a gene trap into the ERdj4 gene resulted in a hypomorphic allele [17]. Although bone marrow cellularity was normal in adult ERdj4^gt/gt^ mice (Figure S1B), CD11b^+^ myeloid cells were significantly increased (Figure 1A–B). In contrast, B220^+^ lymphoid and Ter119^+^ erythroid cells were significantly decreased in the bone marrow of ERdj4^gt/gt^ mice compared to ERdj4^+/+^ controls (Figure 1A–B). Since myeloid, lymphoid and erythroid lineages were all abnormal in ERdj4^gt/gt^ mice, further flow cytometric analysis was performed on early hematopoietic progenitors in the bone marrow. The hematopoietic stem cell (HSC) compartment (LSK, Lin^−^Sca1^+^cKit^+^) was significantly increased in ERdj4^gt/gt^ mice (Figures 1C and S1C). This compartment contains self-renewing, multipotent HSCs, non-self-renewing multipotent progenitors (MPPs) and lymphoid-primed multipotent progenitors (LMPPs) that are primarily committed to myeloid and lymphoid lineages [18], [19]. These populations were distinguished based on Flt3 expression which revealed that LSK cells were elevated due to significantly higher HSCs in the bone marrow of ERdj4^gt/gt^ mice (Figure 1C–D). Common lymphoid progenitors (CLPs), which exclusively generate B and T lymphocytes and natural killer cells, were also increased in ERdj4^gt/gt^ mice compared to controls (Figure 1C–D). Notably, T cell development in the thymus was unaffected by the loss of ERdj4 (Figure S2). Overall, these data suggest that increased myelopoiesis likely resulted from a larger number of HSCs, and that the defect in B lymphopoiesis and erythropoiesis occurred during a later stage of maturation in ERdj4^gt/gt^ mice. Given the impact of the UPR on B cells, subsequent analyses focused to the role of ERdj4 as a UPR effector of B cell development and function.

### The loss of ERdj4 impairs B cell development beginning at the large pre-B cell stage

**Figure 2:**
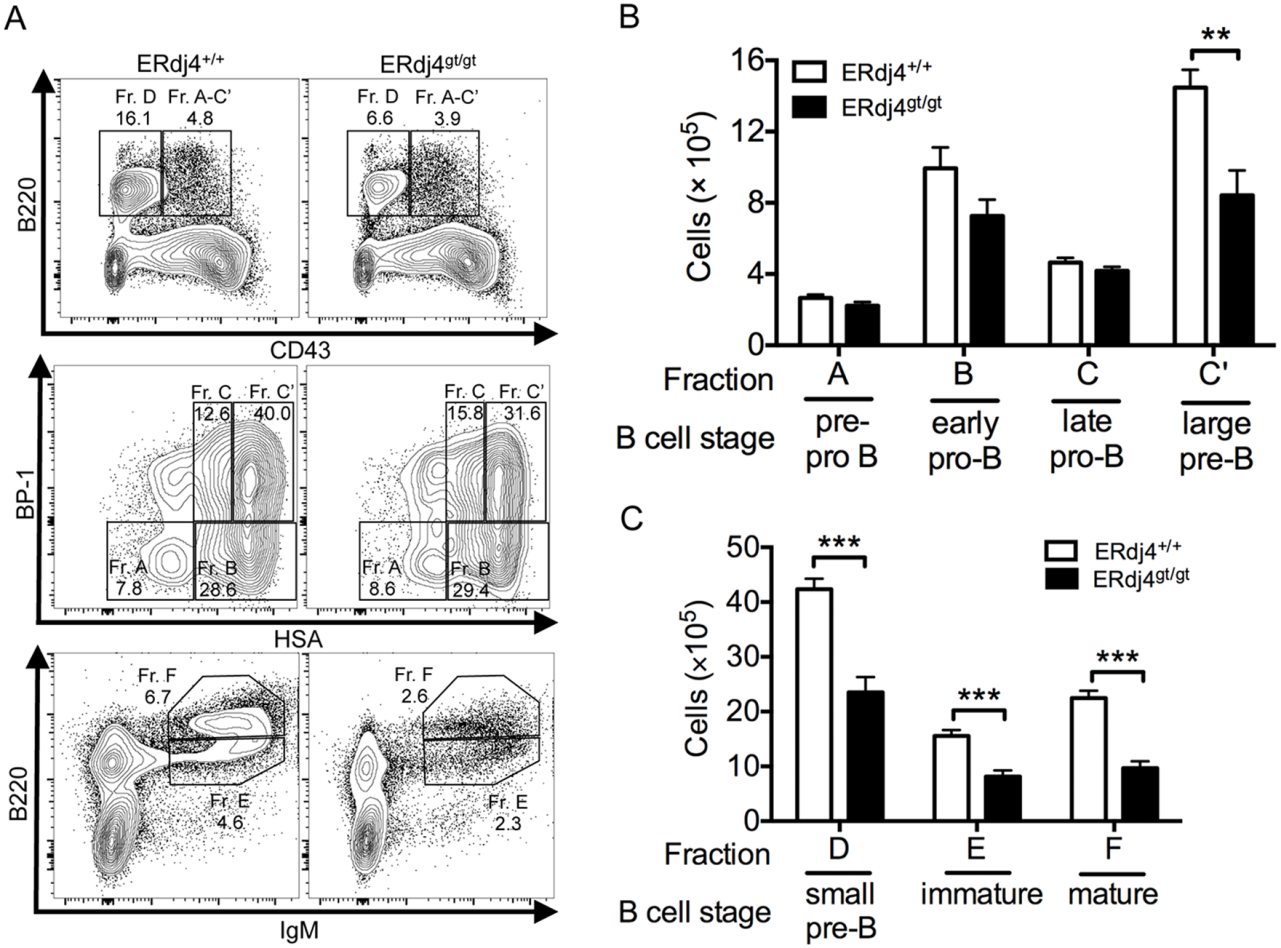
The loss of ERdj4 impairs B cell development beginning at the large pre-B cell stage. (A) Representative contour plots from flow cytometric analyses of B cell development using the Hardy classification scheme. Numbers indicate frequency of the gated population for each genotype. Upper panel, gated on IgM^−^ bone marrow cells. Middle panel, gated on B220^+^CD43^+^ bone marrow cells. Lower panel, gated on total bone marrow cells. Fraction A, B220^+^CD43^+^BP-1^−^HSA^−^; Fraction B, B220^+^CD43^+^BP-1^−^HSA^+^; Fraction C, B220^+^CD43^+^BP-1^+^HSA^low^; Fraction C’, B220^+^CD43^+^BP-1^+^HSA^high^; Fraction D, IgM^−^B220^+^CD43^−^; Fraction E, B220^low^IgM^+^; Fraction F, B220^high^IgM^+^. (B–C) Absolute numbers of Hardy fractions. *n* = 5–8 mice/genotype.

Generation of B lymphocytes from hematopoietic progenitors occurs in sequential stages in the bone marrow. To identify the affected stage(s), B cells were sorted according to the Hardy classification scheme [20]. The earliest B cell precursors were identified by BP-1 and HSA expression on gated B220^+^CD43^+^ cells. Fraction A, which includes pre-pro-B cells, as well as pDCs and natural killer cell precursors, was unchanged in ERdj4^gt/gt^ mice compared to controls (Figure 2A–B). Early and late pro-B cells were also normal in ERdj4^gt/gt^ mice (Fractions B–C, Figure 2A–B); however, there was a significant decrease in large pre-B cells (Fraction C’, Figure 2A–B) and all subsequent stages of B cell development, including small pre-B and immature B cells (Fractions D–E, Figure 2A and C). Mature follicular B cells, which develop contemporaneously in the bone marrow and spleen, were also significantly decreased in ERdj4^gt/gt^ mice compared to controls (Fraction F, Figure 2A and C).

### The defect in B lymphopoiesis is cell autonomous

**Figure 3:**
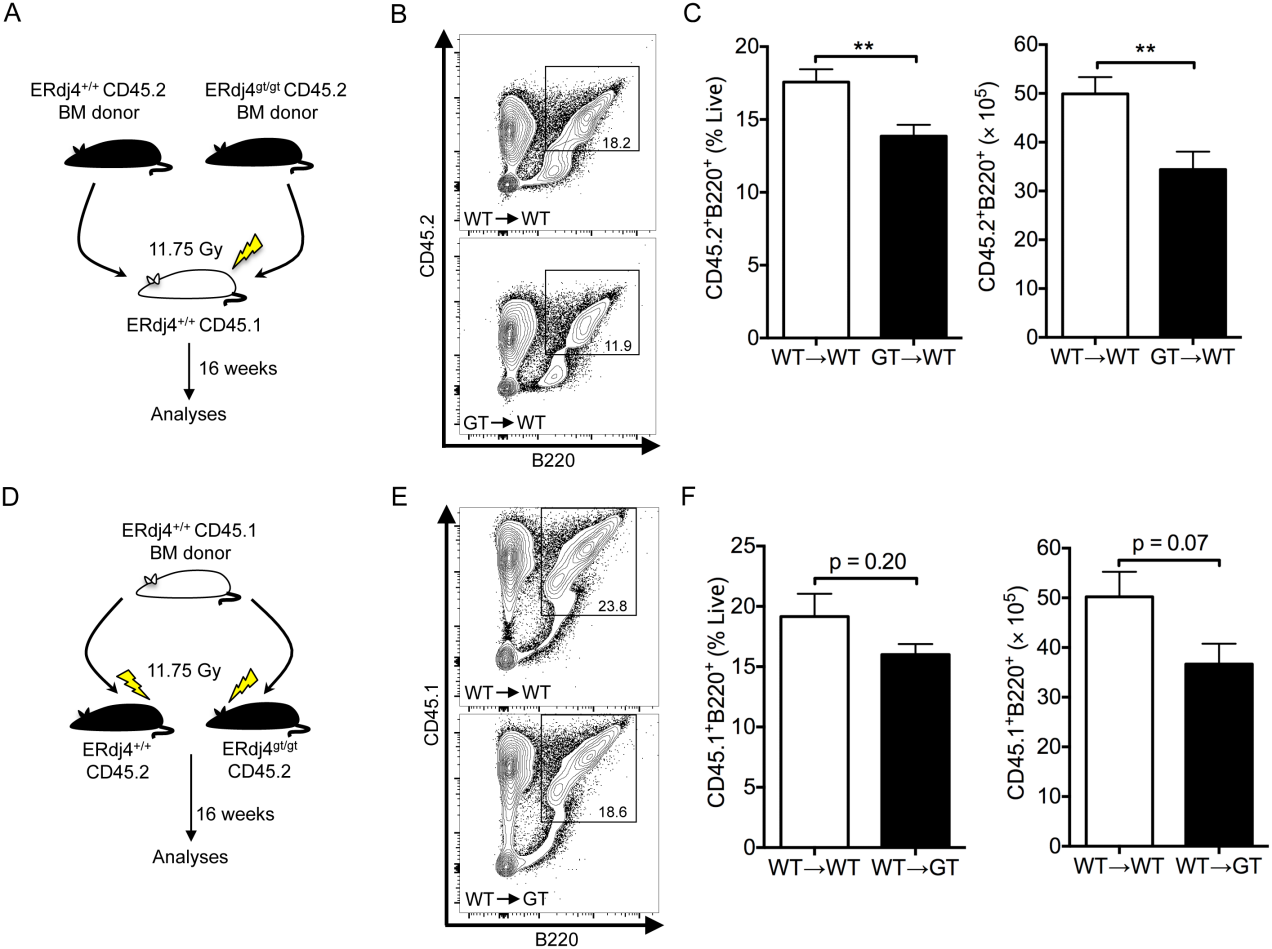
The defect in B lymphopoiesis is cell autonomous. (A, D) Schematics of bone marrow transplants. Recipient mice received two doses of lethal irradiation (7+4.75 Gy) followed by intravenous injection of 5×10^6^ bone marrow cells. (B, E) Representative contour plots from flow cytometric analyses of B cells in the bone marrow 16 weeks post-transplant. Numbers indicate frequency of gated population for each genotype. (C, F) Frequency and absolute number of B cells in the bone marrow as determined by flow cytometry. *n* = 6–8 mice/genotype.

To determine whether the developmental defect was cell autonomous, CD45.2 ERdj4^+/+^ or ERdj4^gt/gt^ bone marrow was transplanted into CD45.1 ERdj4^+/+^ hosts and reconstitution was analyzed 16 weeks after transplantation (Figure 3A). Donor-derived ERdj4^gt/gt^ B cells were significantly reduced in the bone marrow of ERdj4^+/+^ hosts (Figure 3B–C) even in the face of increased numbers of donor HSCs (Figure 1D), suggesting that the effect on B lymphopoiesis was cell autonomous. To confirm a B cell intrinsic defect, CD45.1 ERdj4^+/+^ bone marrow was transplanted into irradiated CD45.2 ERdj4^+/+^ or ERdj4^gt/gt^ mice (Figure 3D). Although the number of donor-derived ERdj4^+/+^ B cells was slightly lower in ERdj4^gt/gt^ mice compared to ERdj4^+/+^ controls, the difference was not statistically significant (Figure 3E–F). Collectively, these data suggest that the defect in B cell development was primarily cell autonomous, but do not exclude the possibility of a minor contribution from the bone marrow microenvironment.

### ERdj4 deficiency reduces survival of B cell progenitors

**Figure 4:**
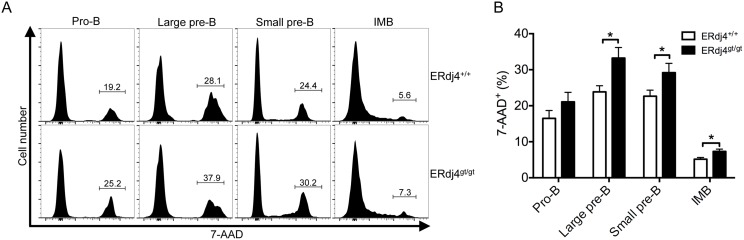
Deficiency of ERdj4 increases cell death during B lymphopoiesis. Developing B cells were stained with 7-AAD and analyzed by flow cytometry. (A) Representative histograms from flow cytometric analyses of B cell progenitors positive for 7-AAD. Numbers indicate frequency of gated population for each genotype. (B) Average frequencies of 7-AAD^+^ B cell progenitors. *n* = 7–8 mice/genotype. Pro-B, IgM^−^CD19^+^B220^+^CD43^+^FSC^low^; Large pre-B, IgM^−^CD19^+^B220^+^CD43^+^FSC^high^; Small pre-B, IgM^−^B220^+^CD43^−^; IMB, B220^low^IgM^+^.

Since developing B cells were reduced in ERdj4^gt/gt^ mice, we assessed proliferation and survival during B lymphopoiesis. Proliferation of developing B cells in the bone marrow was unaffected by ERdj4 deficiency (Figure S3A–B). In contrast, the percentage of 7-AAD^+^ large pre-B, small pre-B and immature B cells was significantly increased compared to controls (Figure 4A–B), consistent with the reduced frequency and number of these B cell progenitors (Fraction C’-E, Figure 2). Since the survival of B cell progenitors depends on pre-BCR and IL-7 receptor α (IL-7Rα) signaling, the expression of these receptors was assessed by flow cytometry. Both the pre-BCR and IL-7Rα were normally expressed on the cell surface of pro-B/large pre-B cells in ERdj4^gt/gt^ mice (Figure S4). Collectively, these data indicate that impaired B lymphopoiesis was the result of reduced survival of B cell precursors in ERdj4^gt/gt^ mice, although the cause of increased cell death remains unclear.

### Follicular B cells are decreased in ERdj4^gt/gt^ mice

**Figure 5:**
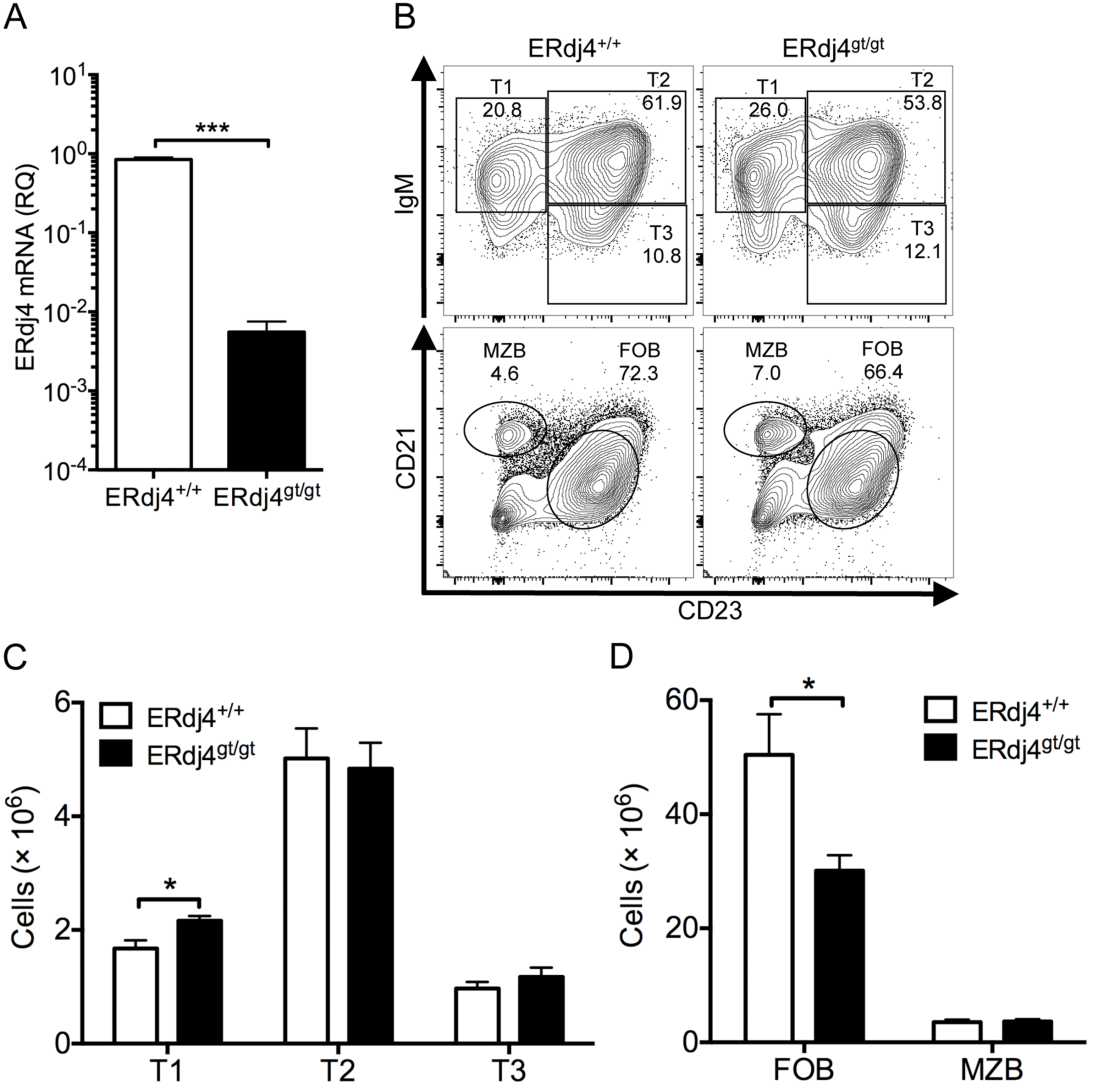
Follicular B cells are reduced in ERdj4^gt/gt^ mice. (A) qRT-PCR of ERdj4 mRNA in spleens isolated from mice; samples were normalized to β-actin. RQ, relative quantitation. *n* = 5 mice/genotype. (B) Representative contour plots from flow cytometric analyses of splenic B cell subsets. Numbers indicate frequency of gated population for each genotype. Transitional B cells (T1, IgM^+^CD23^−^; T2, IgM^+^CD23^+^; T3, IgM^−^CD23^+^) were gated on CD19^+^CD93^+^ cells. Follicular (FOB, CD21^low^CD23^+^) and marginal zone (MZB, CD21^+^CD23^low^) B cells were gated on CD19^+^ cells. (C–D) Absolute numbers of transitional and mature splenic B cell subsets. *n* = 5–6 mice/genotype.

The defect in B cell development in the bone marrow of ERdj4^gt/gt^ mice prompted further analyses of B cells in the periphery. Immature B cells leave the bone marrow and migrate to the spleen where they go through transitional stages (T1 through T2) before differentiation into mature follicular or marginal zone B cells. As in the bone marrow, hypomorphic expression of ERdj4 mRNA was also confirmed in spleen tissue isolated from ERdj4^gt/gt^ mice (Figure 5A). T1 cells, the most recent bone marrow emigrants, were modestly but significantly increased, while T2 and T3 cells were unaffected by ERdj4 deficiency (Figure 5B–C). Marginal zone B cells were normal, but follicular B cells were significantly reduced in ERdj4^gt/gt^ mice compared to controls (Figure 5B and D). The latter finding is consistent with significantly lower mature B cells in the bone marrow (Fraction F, Figure 2A and C). The absolute number of follicular B cells is influenced by production in the bone marrow and spleen, homeostatic proliferation and survival. To identify whether ERdj4 affected proliferation, ERdj4^+/+^ and ERdj4^gt/gt^ mice were pulsed with BrdU for 24 hours and splenic B cell subsets were analyzed for BrdU incorporation by flow cytometry. The frequency of BrdU^+^ transitional, follicular and marginal zone B cells was not affected by ERdj4 deficiency (Figure S5A). Moreover, splenic B cell survival was also unaffected by ERdj4 deficiency (Figure S5B). These data suggest that the lower number of follicular B cells is unrelated to defects in proliferation or survival but is rather the result of impaired development in the bone marrow of ERdj4^gt/gt^ mice, consistent with the findings of Cariappa and Lindsley [21], [22].

### ERdj4 deficiency enhances immunoglobulin isotype switching

**Figure 6:**
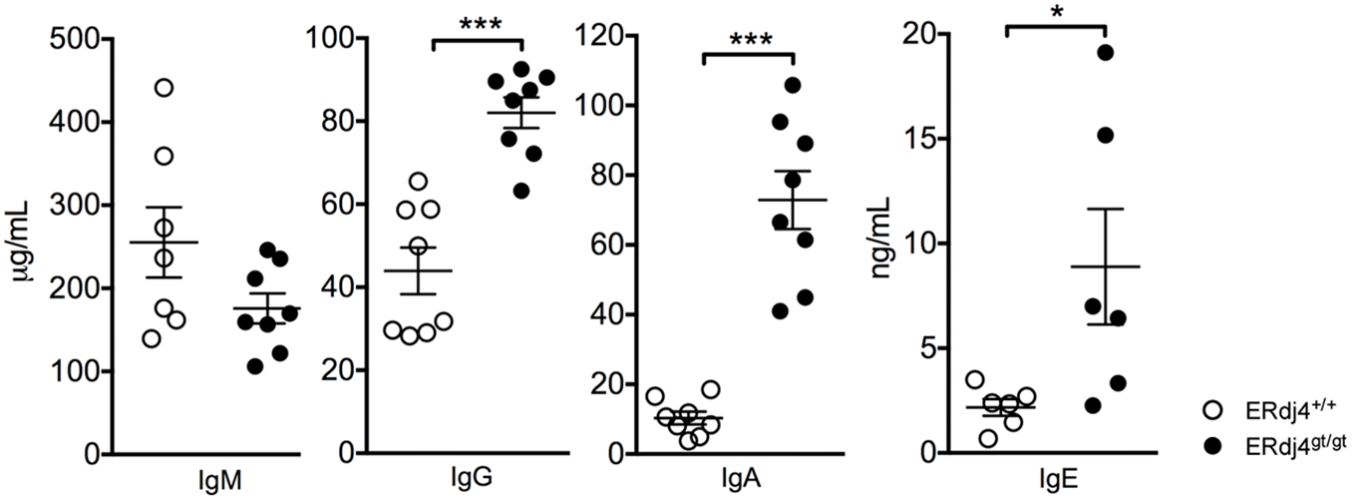
Basal immunoglobulins are increased in ERdj4^gt/gt^ mice. Basal serum immunoglobulin levels were quantitated in ERdj4^+/+^ (open circles) and ERdj4^gt/gt^ (closed circles) mice by isotype-specific ELISA. *n* = 6–8 mice/genotype.

**Figure 7:**
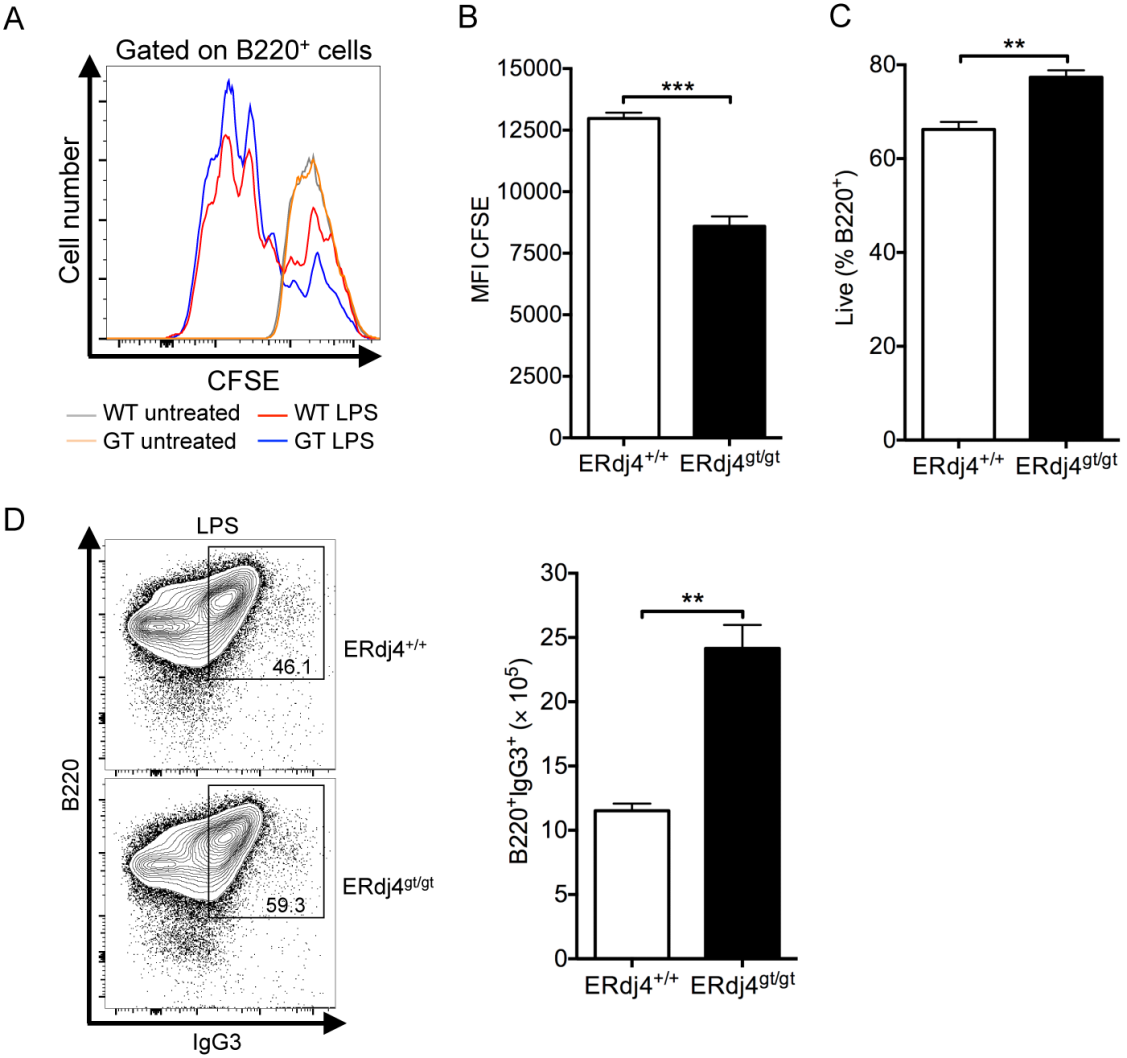
ERdj4 deficiency enhances proliferation, survival and immunoglobulin isotype switching of LPS-treated B cells. (A) CFSE dilution of B220^+^ cells treated with or without LPS. *n* = 3 samples/group. (B) Mean fluorescence intensity (MFI) of CFSE in B220^+^ cells treated with LPS. *n* = 3 samples/group. (C) Frequency of live B220^+^ cells treated with LPS. *n* = 3 samples/group. (D) Left panel, representative contour plots from flow cytometric analyses of B220^+^IgG3^+^ cells following treatment with LPS. Numbers indicate frequency of gated population for each genotype. Right panel, absolute numbers of B220^+^IgG3^+^ cells. *n* = 3 samples/group.

Expression of ERdj4 is regulated by XBP1, a transcription factor required for the production and secretion of antibodies by plasma cells [8]. The frequency and number of plasma cells was normal in ERdj4^gt/gt^ mice (Figure S6A), similar to findings in mice with XBP1-deficient B cells [11], [12]. To assess whether loss of ERdj4 altered the function of B cells, basal immunoglobulins were quantitated in the serum of ERdj4^gt/gt^ mice. While levels of serum IgM were normal, IgG, IgA and IgE were significantly increased in ERdj4^gt/gt^ mice (Figure 6), suggesting that ERdj4 negatively influences B cell activation and/or class switch recombination. To examine these possibilities, resting splenic B cells isolated from ERdj4^+/+^ or ERdj4^gt/gt^ mice were stimulated with LPS to induce plasma cell differentiation and isotype switching to IgG3 (and IgG2b). Consistent with previous findings [9], [12], ERdj4 mRNA was significantly increased in ERdj4^+/+^ B cells stimulated with LPS compared to untreated controls (Figure S6B). ERdj4 mRNA was not detected in resting splenic B cells isolated from ERdj4^gt/gt^ mice; however, a very small amount of ERdj4 mRNA was detected following LPS stimulation, and expression was three logs lower relative to wild-type controls (Figure S6B). Dilution of CFSE was increased and CFSE mean fluorescence intensity was decreased in ERdj4^gt/gt^ B cells stimulated with LPS (Figure 7A–B), consistent with a significant enhancement of cell proliferation. Further, the percentage of live ERdj4^gt/gt^ B cells was significantly higher following LPS stimulation (Figure 7C). Consistent with these findings, the frequency and number of permeabilized B cells expressing IgG3 were significantly increased in LPS-treated cultures deficient in ERdj4 compared to controls (Figure 7D). Together, these results revealed an unexpected negative regulatory role for ERdj4 in B cell activation and non-specific antibody production.

### Impaired T cell-dependent antibody responses in ERdj4^gt/gt^ mice

**Figure 8:**
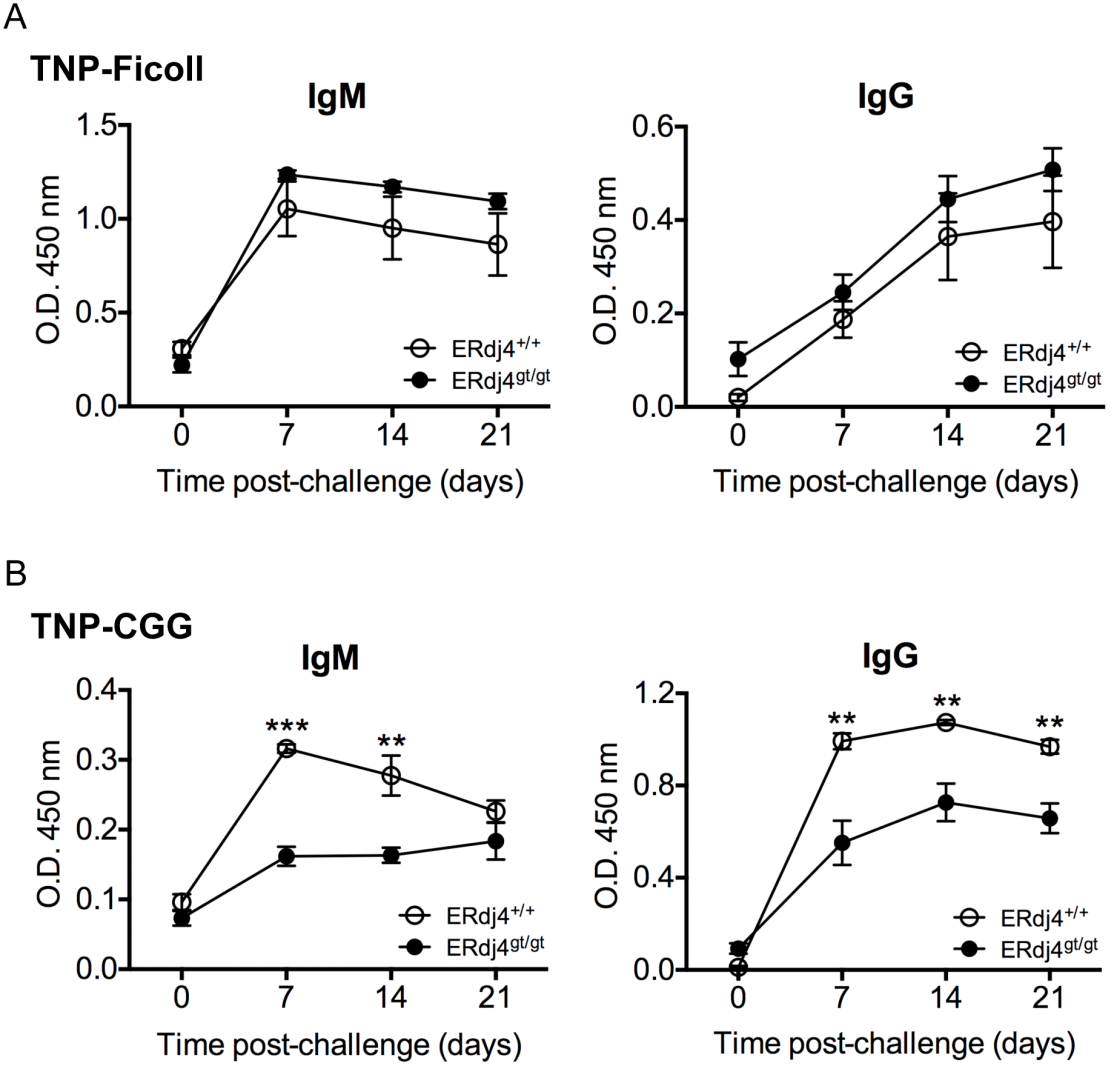
Impaired T cell-dependent antibody responses in ERdj4^gt/gt^ mice. (A–B) Mice were immunized with either TNP-Ficoll (A, 50 µg/100 µl i.p.) or TNP-CGG (B, 100 µg/200 µl i.p.). Plasma TNP-specific IgM and IgG antibodies were quantitated at baseline and weekly post-immunization. *n* = 4–5 mice/group.

Although isotyped-switched antibodies were increased at baseline in ERdj4^gt/gt^ mice, it was unclear whether ERdj4^gt/gt^ B cells were capable of generating antigen-specific antibody responses. To address this question, antigen-specific immunoglobulins were assessed in mice challenged with either T cell-independent (TNP-Ficoll) or -dependent (TNP-CGG) antigen. ERdj4^gt/gt^ mice mounted a normal TNP-specific IgM and IgG response to TNP-Ficoll (Figure 8A), consistent with ability of ERdj4^gt/gt^ B cells to generate immunoglobulins in response to LPS (Figure 7D), another T cell-independent antigen. In contrast, ERdj4^gt/gt^ mice failed to generate a robust antibody response to T cell-dependent antigen; TNP-specific IgM was significantly decreased on days 7 and 14, while TNP-specific IgG was significantly reduced on days 7, 14 and 21 following immunization with TNP-CGG (Figure 8B). Although modest, the IgM antibody response to TNP-CGG was sustained in mice deficient in ERdj4 whereas the IgM antibody response in control mice peaked at day 7 and declined steadily thereafter (Figure 8B). Collectively, these data suggest that the chaperone activity of ERdj4 is required for T cell-dependent antibody responses.

## Discussion

ERdj4 is regulated by the UPR to facilitate the removal of unfolded/misfolded proteins from the ER lumen for degradation by the proteasome. Although ERdj4 is clearly required for ERAD of specific terminally misfolded proteins, emerging evidence suggests that it may also play a more general role in productive protein folding in highly metabolic cells. Mice deficient in ERdj4 exhibit constitutive ER stress associated with defects in growth, development and metabolism [17]. The current study extends these findings by demonstrating that hypomorphic expression of ERdj4 leads to alterations in hematopoiesis and lymphocyte function.

ERdj4 deficiency in adult mice resulted in a greater number of HSCs in the bone marrow. Although this finding indicates that the loss of ERdj4 disrupts HSC quiescence, the subsequent increase in CLPs and myeloid cells suggests that HSCs maintain the ability to differentiate. The larger number of HSCs is likely the result of an unsuccessful attempt to compensate for impaired production of both lymphoid and erythroid lineages in ERdj4^gt/gt^ mice. HSCs are regulated by cytokines and growth factors produced by cells in the bone marrow niche [23]: the reduction in B lymphocytes and/or erythrocytes could lead to stromal cell signaling that stimulates hematopoiesis. The observation that ERdj4^gt/gt^ mice have a defect in erythropoiesis is a potentially important finding that remains to be explored. Of interest, BiP is highly expressed during terminal differentiation of erythrocytes to promote folding and translocation of membrane glycoproteins [24]. Given the known function of ERdj4 in binding/presenting substrate proteins to BiP, it is likely that the BiP/ERdj4 chaperone pair is required for maturation of key, although as yet unidentified, clients during differentiation of erythroid cells. Likewise, BiP/ERdj4 may also play an important role in B cell maturation and function (discussed below).

Despite an increase in early hematopoietic progenitors, B lymphopoiesis was compromised at the transition from pro-B to large pre-B cells. This stage represents a critical checkpoint where signaling through the pre-BCR ensures successful gene rearrangement and expression of immunoglobulin heavy chain. This results in the differentiation and expansion of large pre-B cells, a process that is dependent on IL-7Rα signaling. Deficiency in the enzymes responsible for immunoglobulin gene rearrangement (Rag1/2), the pre-BCR complex, and IL-7Rα, severely disrupt development at this stage [25]. Several genes involved in ER homeostasis and/or function also influence the pre-B cell developmental checkpoint. Similar to findings in the current study, deficiency of the UPR signal transducer IRE1α reduced the number of pre-B cells in the bone marrow. This defect resulted from impaired heavy chain gene rearrangement, associated with reduced expression of Rag1, Rag2 and terminal deoxynucleotidyl transferase [3]. However, it is unlikely that ERdj4 plays a significant role in immunoglobulin heavy chain synthesis as the pre-BCR is normally expressed in ERdj4^gt/gt^ mice. An alternative explanation for the lymphopoietic defect in ERdj4^gt/gt^ mice is suggested from studies of the ER chaperone gp96, an HSP90 family member. gp96 functions both in ERAD [26] and in the productive folding of multiple integrins associated with the hematopoietic system [27]–[29]. Deficiency of gp96 in HSCs impaired the transition from late pro-B to large pre-B cells, similar to findings in ERdj4^gt/gt^ mice. The developmental block was attributed to a defect in stromal cell contact, likely resulting from the loss of integrin expression on the cell surface [30]. Thus, while ERdj4 was originally identified as an ERAD-specific BiP cochaperone [14], [15], it may also function in the productive folding of substrates, such as integrins, that are required for normal B lymphopoiesis.

Defective B cell development in ERdj4^gt/gt^ mice was linked to reduced survival of B cell progenitors. Although the exact mechanism(s) underlying stage-specific apoptosis of developing B cells is unclear, at least two pathways are possible. First, ERdj4 deficiency could lead to unresolved ER stress [17] resulting in activation of C/EBP homologous protein (CHOP), induction of BCL2-interacting mediator of cell death (BIM) and apoptosis [31]. Second, ERdj4 could modulate the pro-survival function of BiP in developing B cells. A previous study by Kurisu *et al.* demonstrated that overexpression of ERdj4 (MDG1) prevented cell death induced by ER stress; this cytoprotective effect was dependent on the ERdj4 J domain, which directly associates with BiP to stimulate ATPase activity [32]. BiP promotes cell survival by inhibiting the activation of ER-associated caspases involved in the execution of apoptosis [33]. Therefore, the loss of ERdj4 may attenuate the anti-apoptotic activity of BiP resulting in reduced survival of B cell progenitors.

Approximately 10–20% of immature B cells produced in the bone marrow reach the spleen to develop into mature follicular or marginal zone B cells [34], [35]. The most recent bone marrow emigrants, splenic T1 cells, were significantly elevated in ERdj4^gt/gt^ mice. This unexpected finding could be the result of increased egress from the bone marrow and/or increased survival. Although transitional cells were not impaired, their predecessors, mature follicular B cells, were reduced in ERdj4^gt/gt^ mice. These long-lived, recirculating B cells are maintained in the periphery through homeostatic proliferation [36], which was unaffected by ERdj4 deficiency. Recent studies described development of follicular B cells from immature precursors in the bone marrow [21], [22]. Thus, a likely explanation for the lower number of follicular B cells is reduced maturation in the bone marrow, further underscoring the importance of ERdj4 in B cell development.

Mature B cells terminally differentiate into antibody-secreting plasma cells upon encounter with antigen. This process is heavily dependent on the IRE1α/XBP1 branch of the UPR to increase the secretory apparatus required for antibody production [6]–[9]. ERdj4 is highly upregulated by XBP1 during plasma cell differentiation [9], [12], suggesting a potential role for this chaperone in immunoglobulin synthesis. Unexpectedly, naive ERdj4^gt/gt^ mice exhibited significantly elevated levels of isotype-switched antibodies, including IgG, IgA and IgE. Consistent with these findings, the loss of ERdj4 in B cells enhanced proliferation, survival and isotype switching in response to LPS in vitro. Class switch recombination is regulated by both activating and inhibitory cell surface receptors, including the BCR, CD40, CD22, Toll-like receptors and cytokine receptors [37]. ERdj4 may be required for productive folding and expression of one or more of these receptors. Although the relevant substrates have yet to be identified, these findings suggest that the chaperone activity of ERdj4 is required for negative regulation of B cell activation and isotype switching in the context of non-specific antibody production. However, it is important to note that antigen-specific antibody responses were not augmented in ERdj4^gt/gt^ mice: in fact, mice deficient in ERdj4 exhibited impaired antibody responses to T cell-dependent antigen. The hallmark of T cell-dependent antibody responses are the formation of germinal centers where B cells receive signals from T cells to differentiate into plasma or memory cells [38]. Since ERdj4^gt/gt^ mice were unable to elicit robust T cell-dependent antibody responses, it is likely that ERdj4 plays an important role in the crosstalk between T and B cells. Taken together, these data suggest that ERdj4 is required for mature B cell function, including interaction with T cells.

ERdj4 is a BiP cochaperone that impacts a range of pathways involved in cell homeostasis by promoting maturation or degradation of specific proteins in the ER [13], [17]. The current study supports this concept by demonstrating that hypomorphic expression of ERdj4 in mice leads to reduced survival of large and small pre-B, and immature B cells in the bone marrow, reduced numbers of mature B cells in the bone marrow and spleen, elevated basal levels of isotype-switched antibodies, and reduced antibody responses to T cell-dependent antigen. These findings highlight the importance of ERdj4 for both B cell development and function. Finally, the reduced numbers of erythrocytes in ERdj4^gt/gt^ mice suggests a broader role for ERdj4 in hematopoiesis.

## Supporting Information

Figure S1 **Hematopoietic progenitors in the bone marrow.** (A) qRT-PCR of ERdj4 mRNA in bone marrow cells isolated from adult mice; samples were normalized to β-actin. RQ, relative quantitation. *n* = 5 mice/genotype. (B) Total number of bone marrow cells isolated from the femur and tibia of mice. *n* = 6 mice/genotype. (C) Frequency and absolute number of LSK cells in mouse bone marrow. *n* = 9–11 mice/genotype.
(TIF)

Figure S2 **Thymic T cell development.** (A) Representative contour plots from flow cytometric analyses of T cell development in 3–4 week-old mice. Numbers indicate frequency of gated population for each genotype. (B) Average frequencies of developing T cells in the thymi of mice. *n* = 6 mice/genotype.
(TIF)

Figure S3 **Proliferation of** **developing B cells in the bone marrow.** (A) Representative histograms from flow cytometric analyses of BrdU incorporation in developing B cells. Numbers indicate the frequency of BrdU^+^ cells from pro-B (IgM^−^CD19^+^B220^+^CD43^+^FSC^low^), large pre-B (IgM^−^CD19^+^B220^+^CD43^+^FSC^high^), small pre-B (IgM^−^B220^+^CD43^−^) and immature (B220^low^IgM^+^) subsets. (B) The mean frequency of developing B cells positive for BrdU. *n* = 4–6 mice/genotype.
(TIF)

Figure S4 **Pre-BCR and IL-7Rα expression on pro-B/large pre-B cells.** Representative histograms from flow cytometric analyses of pre-BCR (λ5) and IL-7Rα expression on pro-B/large pre-B cells (CD19^+^B220^+^CD43^+^) in the bone marrow of adult mice. MFI, mean fluorescence intensity. *n* = 4–5 mice/genotype.
(TIF)

Figure S5 **Proliferation and viability of splenic B cells.** (A) Mean frequencies obtained from flow cytometric analyses of BrdU incorporation in splenic B cell subsets. T1–T3, transitional B cells, CD19^+^CD93^+^; FOB, follicular B cells, CD19^+^CD21^low^CD23^+^; MZB, marginal zone B cells, CD19^+^CD21^+^CD23^low^. *n* = 5–6 mice/genotype. (B) Splenocytes were isolated from mice and cultured in complete RPMI medium over time. Viability of CD19^+^ B cells was assessed by flow cytometry using the Fixable Viability Dye eFluor780 (Ebioscience). *n* = 3 mice/group.
(TIF)

Figure S6 **The frequency and number of** **plasma cells in the spleen.** (A) Left panel, Representative contour plots from flow cytometric analyses of plasma cells in the spleens of adult mice. Numbers indicate frequency of the gated population for each genotype. Right panel, absolute number of plasma cells. *n* = 6 mice/genotype. (B) qRT-PCR of ERdj4 mRNA in untreated or LPS-treated B cells isolated from the spleens of adult mice; samples were normalized to 18S rRNA. *n* = 3 mice/genotype. RQ, relative quantitation; u.d., undetected.
(TIF)

Table S1 **Flow cytometry antibodies.**
(TIF)
