# Supplementary figures and images for: The BiP Cochaperone ERdj4 Is Required for B Cell Development and Function
Source: PLoS One. 2014 Sep 15;9(9):e107473. doi: 10.1371/journal.pone.0107473 (PMC4164662; doi:10.1371/journal.pone.0107473)

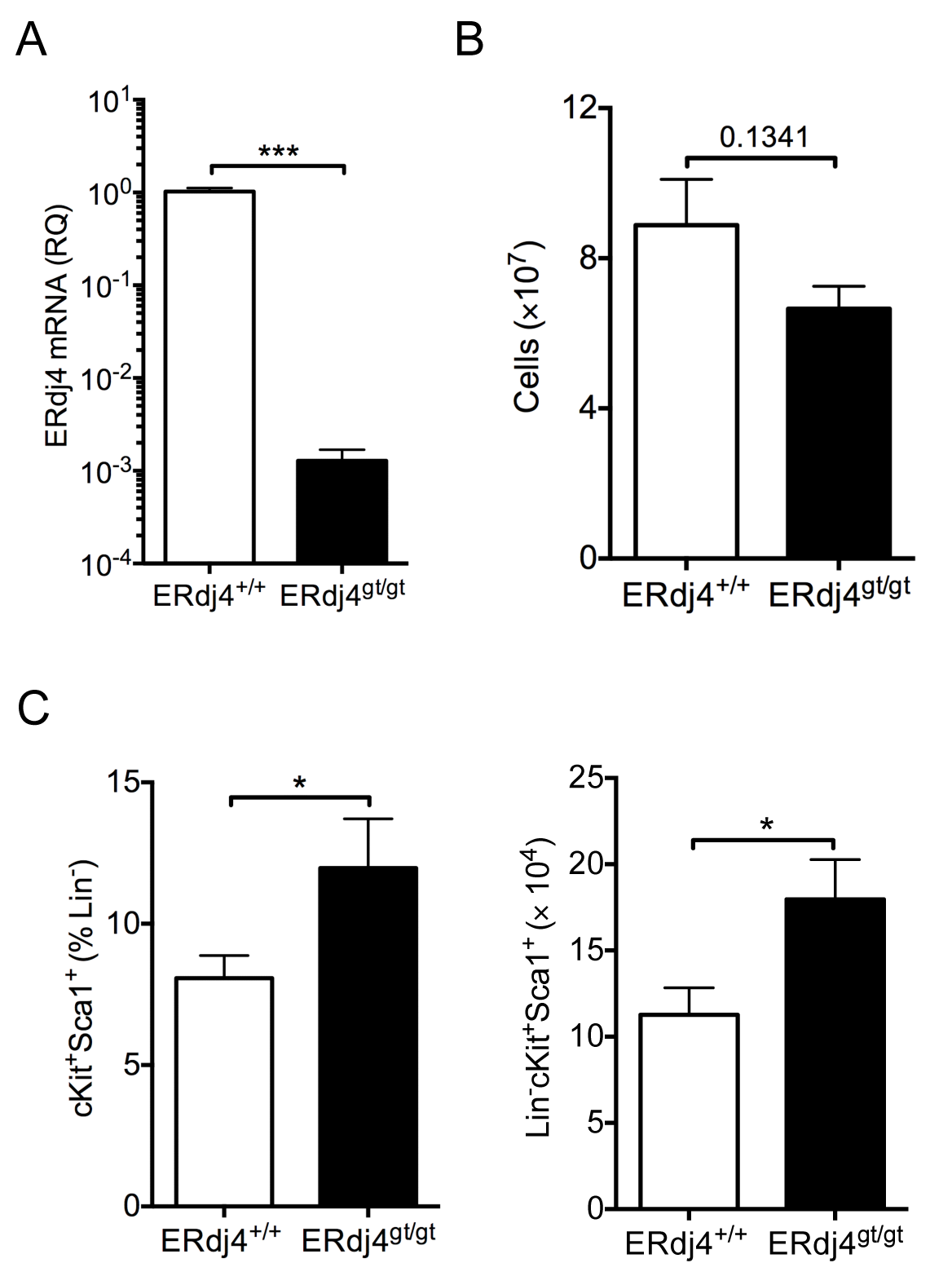

Supplement: Figure S1 — Hematopoietic progenitors in the bone marrow. (A) qRT-PCR of ERdj4 mRNA in bone marrow cells isolated from adult mice; samples were normalized to β-actin. RQ, relative quantitation. n = 5 mice/genotype. (B) Total number of bone marrow cells isolated from the femur and tibia of mice. n = 6 mice/genotype. (C) Frequency and absolute number of LSK cells in mouse bone marrow. n = 9–11 mice/genotype. (TIF) [file pone.0107473.s001.tif]

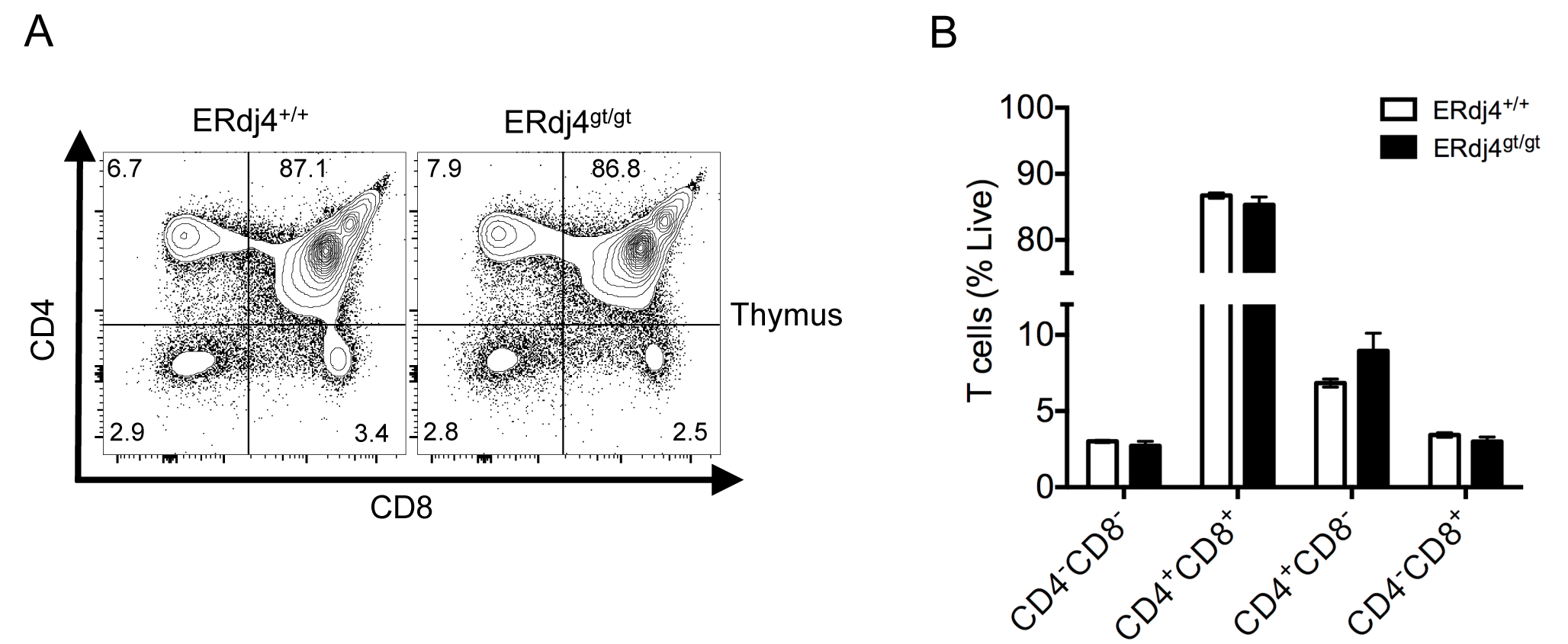

Supplement: Figure S2 — Thymic T cell development. (A) Representative contour plots from flow cytometric analyses of T cell development in 3–4 week-old mice. Numbers indicate frequency of gated population for each genotype. (B) Average frequencies of developing T cells in the thymi of mice. n = 6 mice/genotype. (TIF) [file pone.0107473.s002.tif]

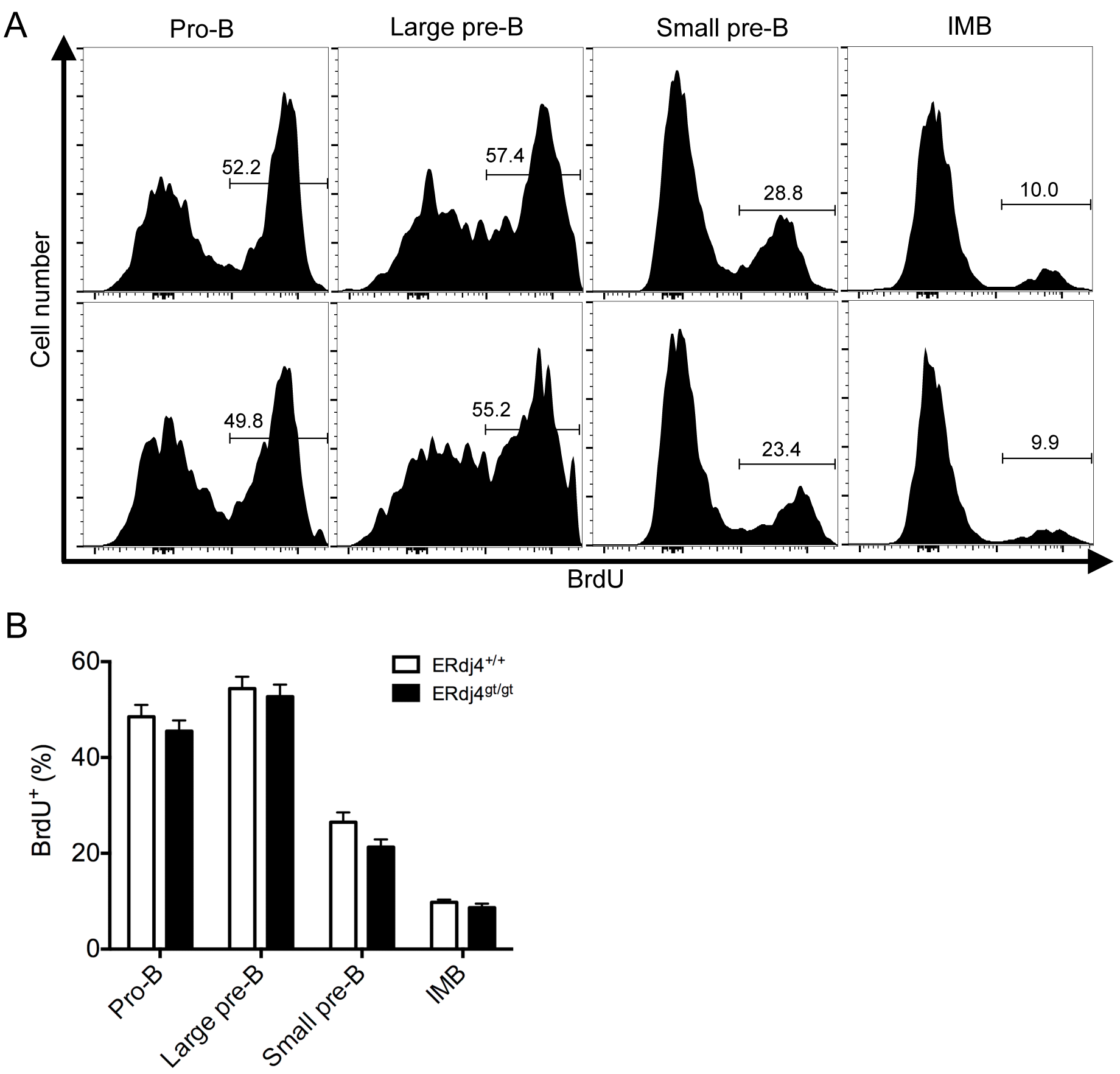

Supplement: Figure S3 — Proliferation of developing B cells in the bone marrow. (A) Representative histograms from flow cytometric analyses of BrdU incorporation in developing B cells. Numbers indicate the frequency of BrdU+ cells from pro-B (IgM−CD19+B220+CD43+FSClow), large pre-B (IgM−CD19+B220+CD43+FSChigh), small pre-B (IgM−B220+CD43−) and immature (B220lowIgM+) subsets. (B) The mean frequency of developing B cells positive for BrdU. n = 4–6 mice/genotype. (TIF) [file pone.0107473.s003.tif]

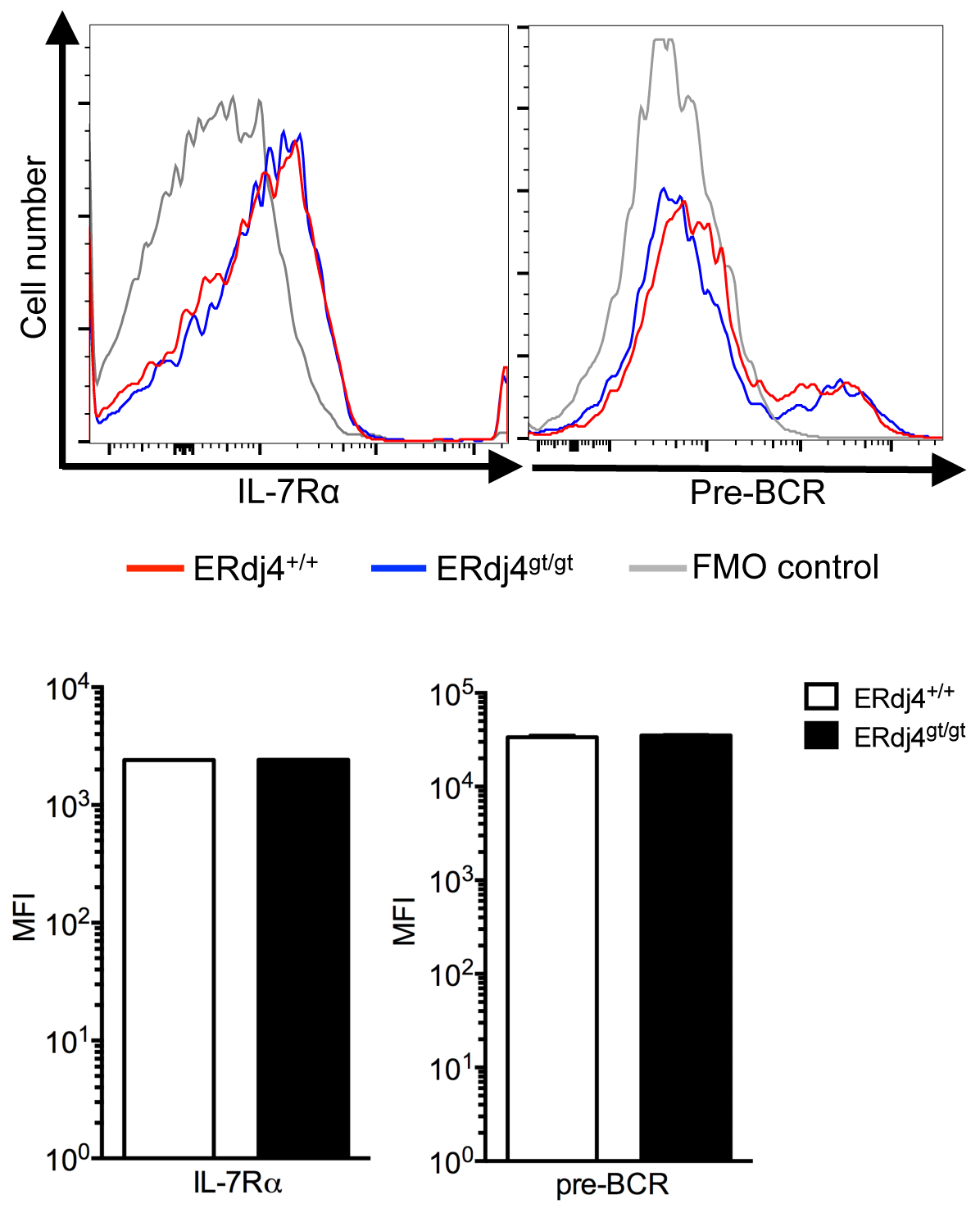

Supplement: Figure S4 — Pre-BCR and IL-7Rα expression on pro-B/large pre-B cells. Representative histograms from flow cytometric analyses of pre-BCR (λ5) and IL-7Rα expression on pro-B/large pre-B cells (CD19+B220+CD43+) in the bone marrow of adult mice. MFI, mean fluorescence intensity. n = 4–5 mice/genotype. (TIF) [file pone.0107473.s004.tif]

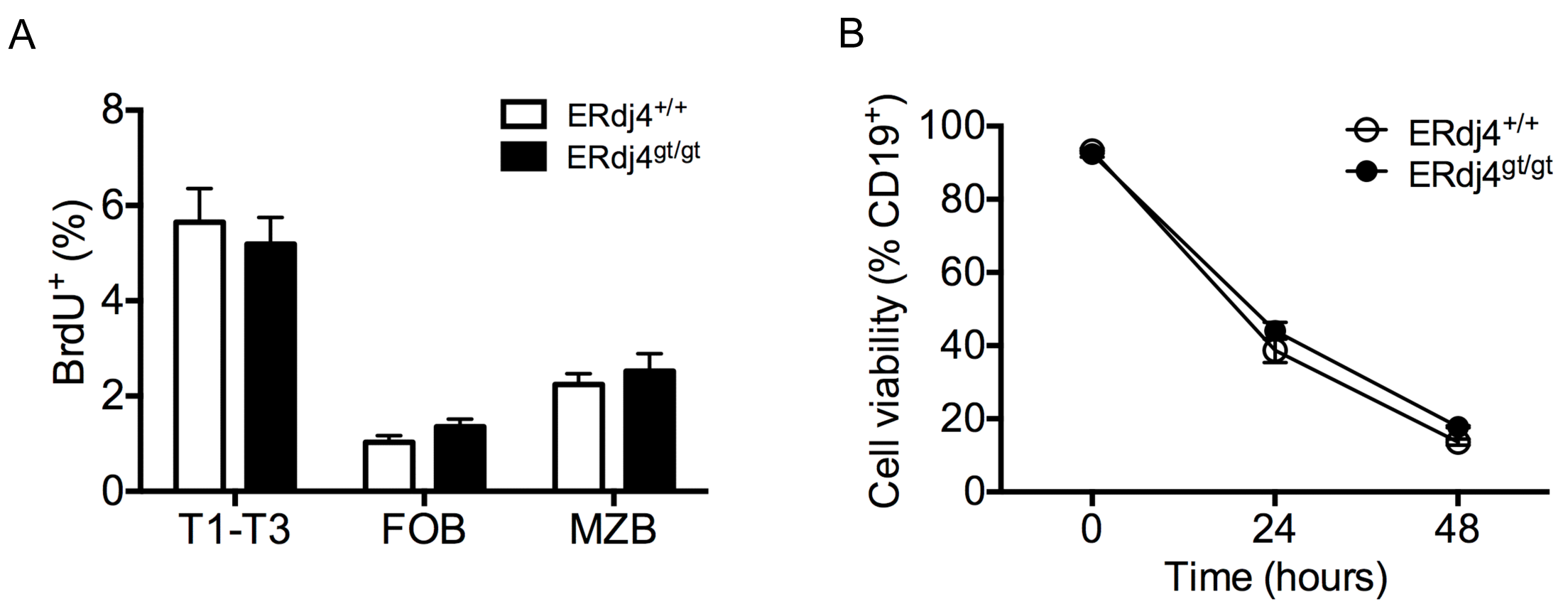

Supplement: Figure S5 — Proliferation and viability of splenic B cells. (A) Mean frequencies obtained from flow cytometric analyses of BrdU incorporation in splenic B cell subsets. T1–T3, transitional B cells, CD19+CD93+; FOB, follicular B cells, CD19+CD21lowCD23+; MZB, marginal zone B cells, CD19+CD21+CD23low. n = 5–6 mice/genotype. (B) Splenocytes were isolated from mice and cultured in complete RPMI medium over time. Viability of CD19+ B cells was assessed by flow cytometry using the Fixable Viability Dye eFluor780 (Ebioscience). n = 3 mice/group. (TIF) [file pone.0107473.s005.tif]

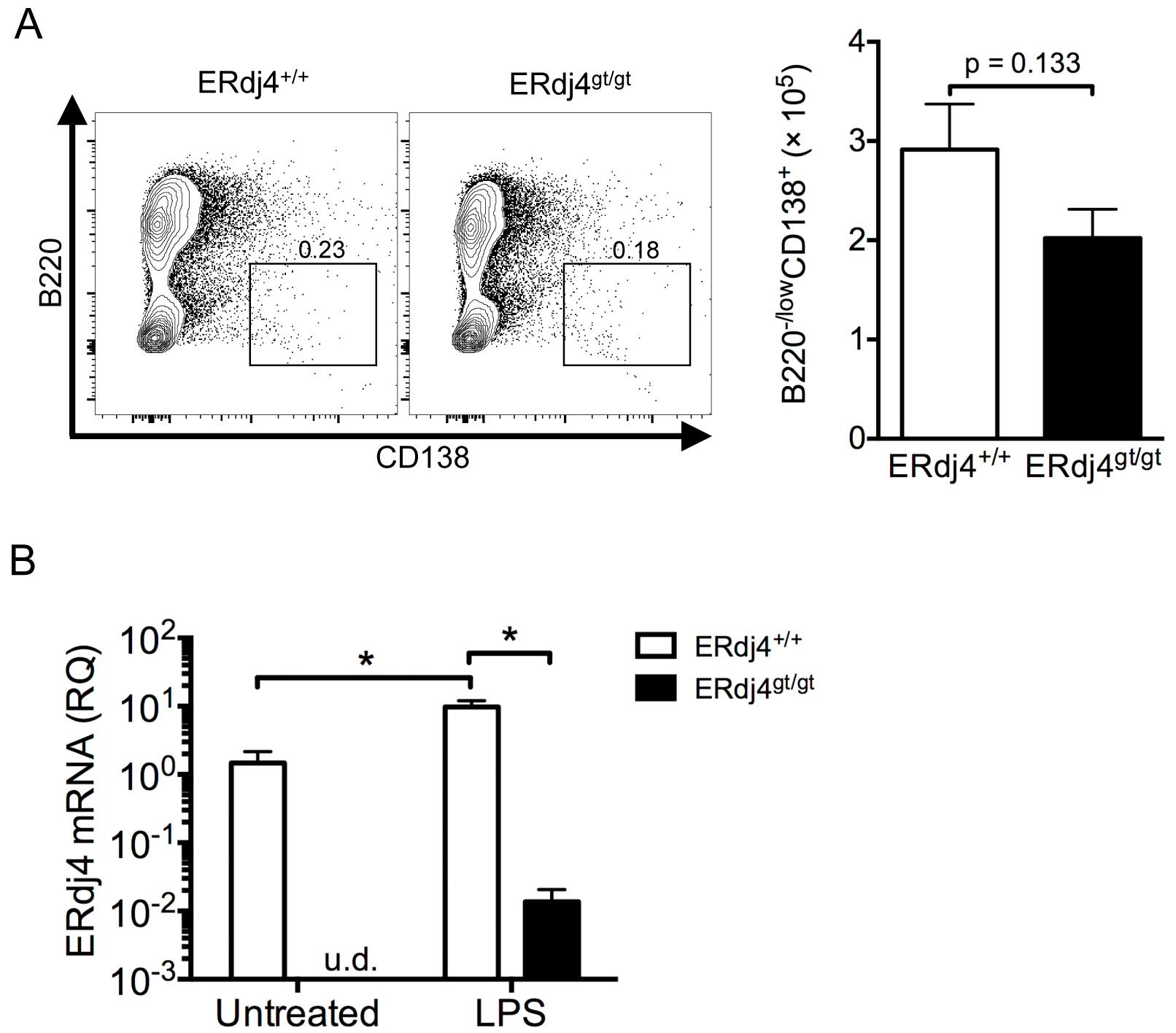

Supplement: Figure S6 — The frequency and number of plasma cells in the spleen. (A) Left panel, Representative contour plots from flow cytometric analyses of plasma cells in the spleens of adult mice. Numbers indicate frequency of the gated population for each genotype. Right panel, absolute number of plasma cells. n = 6 mice/genotype. (B) qRT-PCR of ERdj4 mRNA in untreated or LPS-treated B cells isolated from the spleens of adult mice; samples were normalized to 18S rRNA. n = 3 mice/genotype. RQ, relative quantitation; u.d., undetected. (TIF) [file pone.0107473.s006.tif]

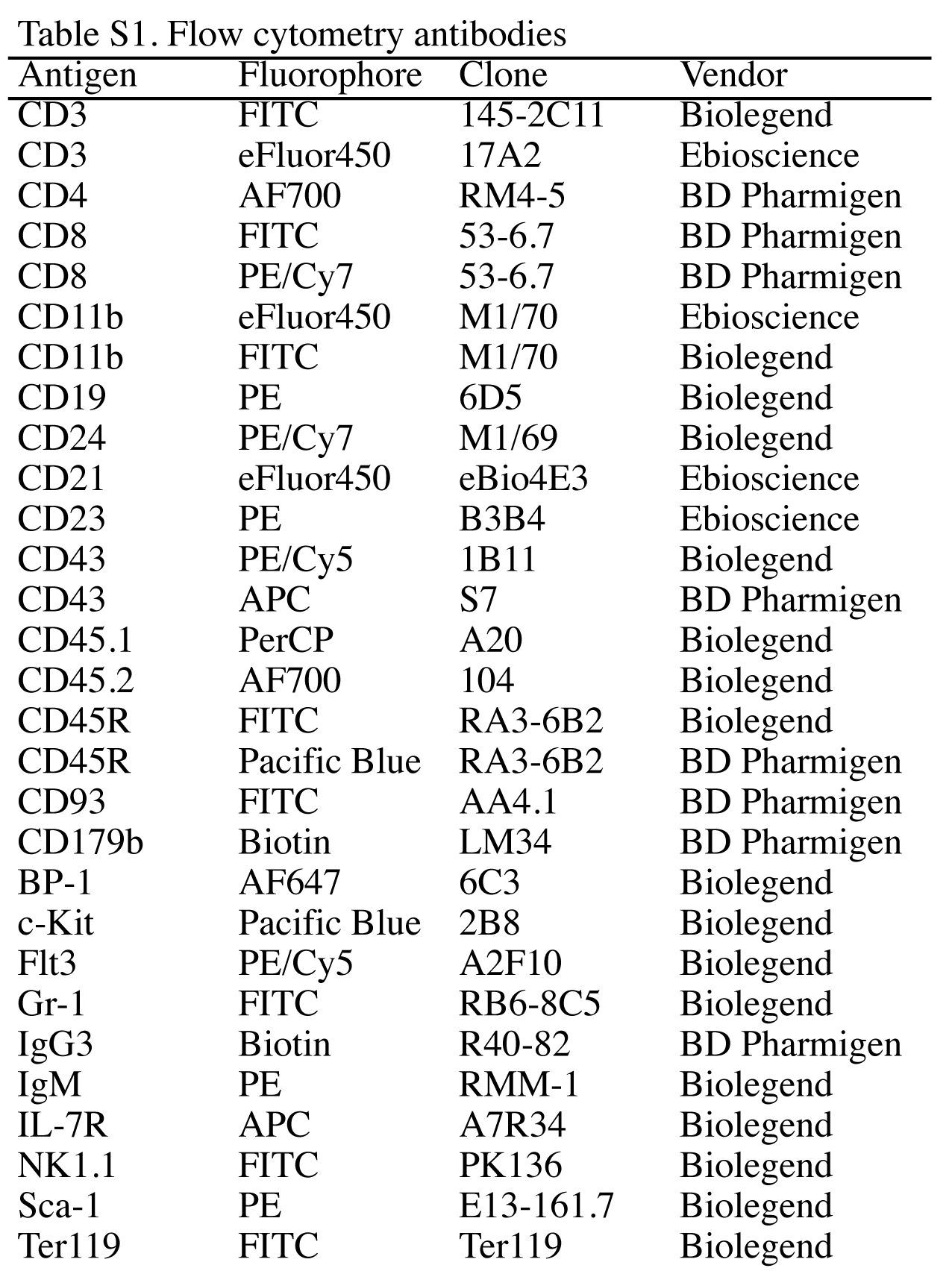

Supplement: Table S1 — Flow cytometry antibodies. (TIF) [file pone.0107473.s007.tif]
